# Individual alpha frequency predicts the sensitivity of time perception

**DOI:** 10.1162/IMAG.a.1263

**Published:** 2026-06-04

**Authors:** Audrey Morrow, Montana Wilson, Michaela Geller-Montague, Sara Soldano, Sabah Hajidamji, Jason Samaha

**Affiliations:** University of California, Santa Cruz, Santa Cruz, CA, United States; University of California, San Francisco, San Francisco, CA, United States; Stanford University, Stanford, CA, United States

**Keywords:** alpha oscillations, individual differences, duration estimation, duration discrimination, time perception

## Abstract

A growing body of research links individual differences in the frequency of alpha-band oscillations to temporal aspects of perception. However, whether the human alpha rhythm is a correlate of time perception itself has remained controversial. This study combined EEG with multiple duration estimation and discrimination tasks in order to evaluate whether individual alpha frequency (IAF) is associated with sensitivity or bias in judging visual durations across a range of peri-second durations (spanning 100 to 1200 ms). In a temporal estimation task, participants (n = 55) reported the duration of a single stimulus between 300–1200 ms. In a temporal discrimination task, participants reported which of two stimuli was longer: a standard (100, 600, or 1200 ms) or comparison (50–150% of the standard). Stimuli also varied in whether their luminance was static or dynamic (varying randomly over time). We found that IAF was significantly related to the variance of duration estimates, a measure of precision (or sensitivity), but not average duration estimates, a measure of bias. Further supporting this relationship, psychometric function slopes obtained from the independent duration discrimination tasks were positively correlated with IAF, particularly for the static stimulus conditions. These effects were largely consistent across the range of stimulus durations tested and held when controlling for participant age. Taken together, these results suggest that IAF may play a role in shaping individual differences in the sensitivity of time perception.

## 1 Introduction

The neural mechanisms underlying the perception of stimulus duration have long intrigued psychologists since, unlike other sensory features, time has no dedicated sensing organ. One view is that intrinsic neural dynamics act as an internal clocking mechanism that contributes to the perceived passage of time (Ivry & Schlerf, 2008). Alpha-band (8–13 Hz) neural oscillations have garnered interest as a potential neural clocking mechanism given their relatively stable rhythmic characteristics, intrinsic genesis, and the fact that individual variation in alpha frequency has been associated with a range of visual and cross-modal temporal integration processes in perception (Cecere et al., 2015; Dou et al., 2022; Drewes et al., 2022; Keil & Senkowski, 2017; Migliorati et al., 2020; Samaha & Postle, 2015; Samaha & Romei, 2024; Venskus & Hughes, 2021). Indeed, early researchers debated the idea that the alpha rhythm reflects the minimum “psychological moment” (Ellingson, 1956; Treisman, 1984; White, 1963). Moreover, individual alpha frequency (IAF) has been shown to correlate with the brain’s temporal response function (TRF) to unit changes in visual features (Chang et al., 2017; Morrow et al., 2026; VanRullen & Macdonald, 2012), further linking alpha to visual processing mechanics over time. Thus, we sought to assess whether the frequency of alpha cycles, as indexed by IAF, relates to individual variation in visual duration perception.

Early research on the link between alpha frequency and time perception (reviewed in van Wassenhove et al., 2019) led to mixed results, with several reports of small-to-medium correlations between IAF and time estimates (Cahoon, 1969; Werboff, 1962), but also null findings (Legg, 1968). However, early work often used durations spanning several seconds up to minutes, which arguably reflect more mnemonic, rather than perceptual, contributions to performance. An important limitation of these earlier studies is that the measures of time perception that were used often did not distinguish between bias (over-/under-estimation of time) and sensitivity/precision (the ability to reliably judge or distinguish intervals). If alpha cycles are, indeed, underlying the perception of “moments”, an association between IAF and bias would favor an account where more frequent moments correspond to a longer perceived duration. On the other hand, an association between IAF and sensitivity would suggest that more frequent moments simply provide temporal discrimination mechanisms with more information per unit of time, allowing for a greater ability to represent and distinguish durations the higher the IAF.

Some recent work linking time perception and alpha activity has assessed bias and sensitivity using shorter, peri-second durations, although methods and findings remain inconsistent. A study by Milton and Pleydell-Pearce (2016) used a two-interval forced choice (2IFC) temporal discrimination task to explore links between IAF and prestimulus alpha phase on bias and sensitivity. They found that IAF and phase were related to bias in perception of durations around 400 ms, as measured by the point of subjective equality (PSE), but IAF was not related to sensitivity, as measured by the slope of individual psychometric functions. Another recent study by Mioni et al. (2020) used tACS at 2 Hz +/- IAF during a temporal generalization task and found that stimulation above (below) IAF produced a bias toward longer (shorter) judgments of visual stimuli compared to a 500 ms memorized standard. However, they did not find an effect of stimulation on the precision of perceptual judgments. In contrast to these results on bias, Mokhtarinejad et al. (2024) used a very similar temporal generalization task to Mioni et al. (2020), except with a 1000 ms standard, and found a positive correlation between IAF and precision, but not bias. However, they also found no effect of tACS stimulation above or below IAF. One possible source of the variability in these findings is the use of relatively small sample sizes for individual differences research. The three studies reviewed above used samples between 15 and 24 participants, leaving open the possibility that discrepant results reflect sampling error. Recently, Frisoni et al. (2025) evaluated a larger sample of 50 participants in a 2IFC task with 100 ms and 500 ms standards, and found support for the role of IAF in sensitivity, but not bias, of duration perception. We sought to expand on these findings by testing a similarly large EEG sample across two types of duration perception tasks and a range of standard durations, comparable to what has been most commonly used in the duration perception literature.

In this study, we specifically focused on IAF as a measure of individual differences in visual time perception, given the associations between alpha and visual processing in the literature as well as the ability to observe clear peaks in the alpha-band range, which is more difficult in other frequencies (Donoghue et al., 2020). We derived measures of both sensitivity (precision) and bias in peri-second temporal estimation, which requires observers to judge stimulus durations relative to an internal reference, and in temporal discrimination, which requires a comparative judgment of two external stimulus durations. The discrimination task introduced a dynamically modulating stimulus, known to induce alpha-band impulse responses in the visual system (VanRullen & Macdonald, 2012), with the hypothesis that the dynamic stimulus condition may provide a clearer association between IAF and duration discrimination. We identified IAF from eyes-closed resting-state EEG recordings and observed positive correlations between IAF and precision in the duration estimation task and sensitivity in the discrimination task, but no relationship with bias at the individual level. In addition, we observed an interaction between IAF and stimulus type that suggested, surprisingly, a stronger relationship between IAF and performance for the static stimulus condition compared to the dynamic condition. While our sample consisted of young adults, where we might not yet expect to see differences in IAF (Quinn et al., 2024), age-related differences in duration perception task performance may still occur and were, indeed, observed in our sample. However, when controlling for age in our analyses, the correlation between IAF and sensitivity holds. Our findings suggest that individuals with higher alpha frequencies have more sensitive time perception, though stimulus features may moderate this relationship.

## 2 Method

### 2.1 Participants

This study was approved by the University of California Santa Cruz (UCSC) Institutional Review Board. Fifty-five participants with a mean age of 21.46 (SD = 5.16, range: 18–35) were recruited from UCSC’s online participant portal (Sona Systems) and from the Samaha Lab. This sample size was chosen to achieve 90% power to detect a correlation in the range suggested by a recent meta-analysis of the correlation between IAF and temporal binding measures (mean r ~ 0.45) (Samaha & Romei, 2024). The study consisted of 2 days of testing; the first day of testing took no more than 1.5 hours and involved the completion of questionnaires and behavioral tasks. Participants received 1.5 research credits and a $10 Amazon gift card. The second day of testing, which had to be completed within 12 days of the first, took no more than 3.5 hours and involved the completion of several duration perception tasks with simultaneous EEG recording. Participants were rewarded with 3.5 research credits and a $30 Amazon gift card. The sample consisted of 68.52% of participants who identified as female, 24.07% who identified as male, and 7.41% who identified as non-binary. The participant sample was 45.45% White/Caucasian, 25.45% Asian (35.71% did not specify further, but of those who did specify, Indian-identifying participants made up 14.29% and those who identified as either Chinese, Cambodian, Filipino, Japanese, Persian, Taiwanese, or Vietnamese each made up 7.14%), 20% Hispanic/Latino, and 9.09% multiracial (20% Black/Southeast Asian, 20% White/Indian, 20% White/Filipino, 20% White/Taiwanese, and 20% White/Chinese). Participants all reported having normal or corrected-to-normal vision.

### 2.2 Duration estimation task

**Fig. 1.**
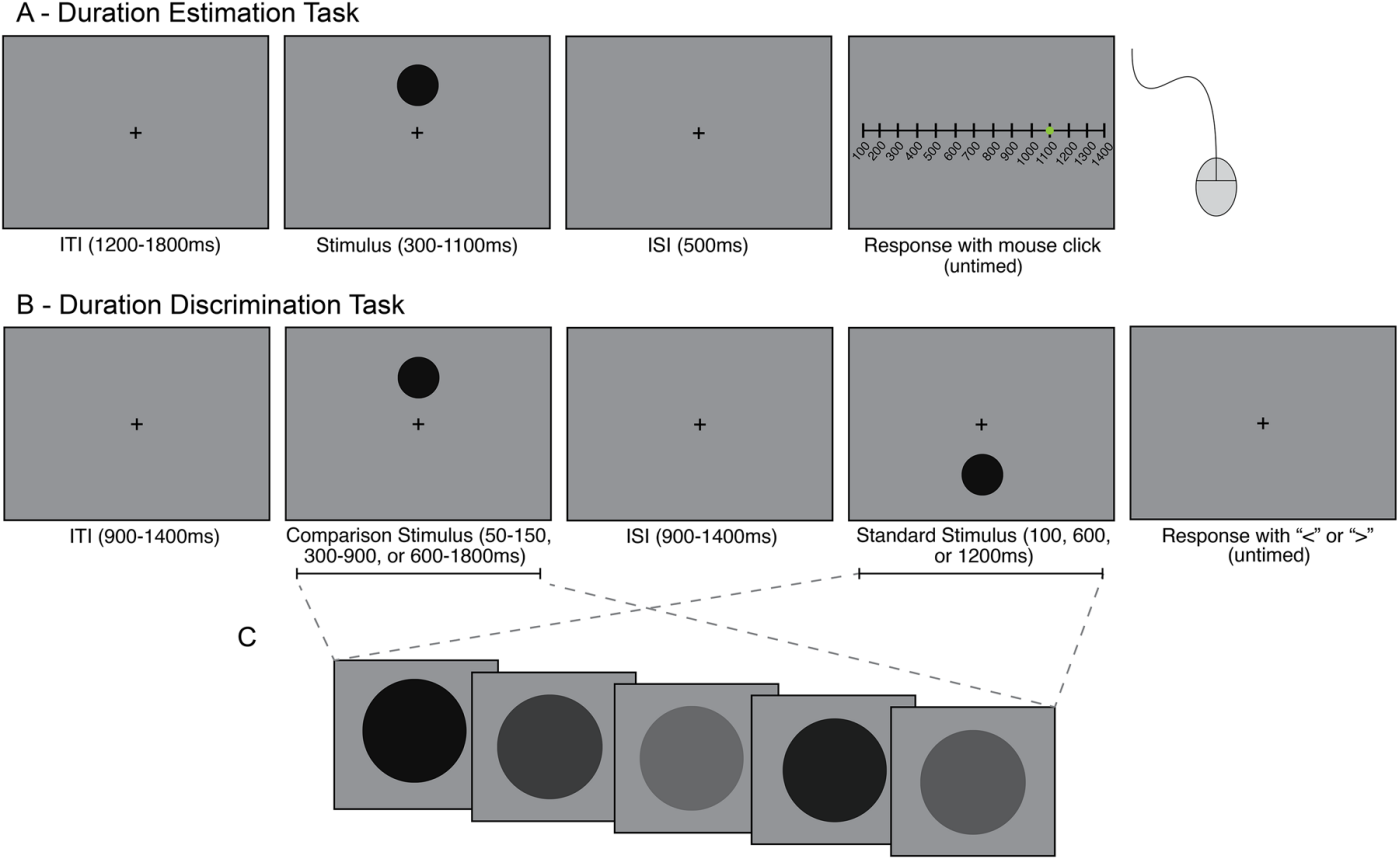
Diagram of duration perception tasks. (A) Duration estimation task. Participants were shown a dot of variable duration either above or below the central fixation and reported their estimate of the duration of the dot via mouse click along a slider of possible durations. (B) Duration discrimination task. Participants were presented with a dot above fixation, followed by a dot below fixation, or vice versa (dot presentation alternated by block) and reported, via button press, whether the first or second dot was longer. (C) A schematic of the dynamic stimulus condition. The luminance value of the stimulus in the dynamic condition varied randomly between black and gray on every screen refresh (120 Hz) throughout the stimulus presentation.

In the duration estimation task participants provided estimates of the duration of a simple visual stimulus (Fig. 1A). In both the practice and experimental blocks, participants observed a dark gray dot presented on a medium gray background at either 3 degrees of visual angle (DVA) above or below the central fixation. The dot duration was pseudo-randomly chosen between 300–1100 ms in 100 ms intervals with equal probability. Participants estimated each stimulus duration using a slider, controlled via a computer mouse, that displayed a number line of possible durations, ranging from 100–1400 ms in 100 ms intervals. The slider provided numbers outside of the range of actual durations to reduce bias when reporting the shortest and longest possible durations. The current value was displayed on the screen in numerical values and was represented on the slider by a green dot, which appeared at a random starting location on each trial. The inter-trial interval (ITI) ranged between 1.2 and 1.8 s after the response was input. The practice block was 48 trials long and provided feedback displaying the actual stimulus duration if participants were incorrect in their estimate or “Correct!” if participants were correct. The two experimental blocks lacked feedback and were 90 trials long with each possible duration presented 10 times, totaling 180 trials.

### 2.3 Duration discrimination task

The duration discrimination task was a two-interval forced choice (2IFC) design in which participants were asked to report which of two visual stimuli they perceived to be longer in duration (Fig. 1B-C). This task had two conditions: a “static” condition, where the stimulus was the same gray dot as presented in the duration estimation task, and a novel “dynamic” condition where the dot varied randomly in luminance between medium gray (0.5 luminance) to black (0 luminance) on every monitor refresh (120 Hz). To make the dynamically modulating stimulus, we created random luminance sequences for each dot presentation on each trial, and then normalized the sequence amplitude in the Fourier domain before performing an inverse Fourier transform to ensure the dot had equal energies at all frequencies. Besides the introduction of a flickering dot, the dynamic condition was identical to the static condition. Unfortunately, we were unable to implement this condition in the estimation task due to time constraints of the study. The order of discrimination conditions was counterbalanced across experimental sessions.

In both duration discrimination conditions, participants observed the first dot either 3 DVA above or below the central fixation, and then a second target on the opposite side of fixation (to avoid any adaptation effects), with the presentation order counterbalanced across blocks so that participants could always expect the location of the first dot as well as the second dot. In other words, whether the first stimulus was presented above or below fixation alternated from block to block but was held constant across the block. For all blocks, one target (the “standard”) was presented for a standard duration of 100, 600, or 1200 ms, and the other target (the “comparison”) was presented for a fraction of the standard duration (either 50%, 70%, 90%, 110%, 130%, or 150% of the standard duration). In this way, the comparison was always proportional to the standard, scaling the comparison durations to be longer for the longer standard durations and shorter for the shorter standards in order to equate difficulty for the different standard durations as predicted by Weber’s Law (Haigh et al., 2021). There was a total of 30 presentations of each standard and comparison pair for each task condition. After participants responded with a button press to indicate whether the first (“<”) or the second (“>”) target was longer in duration, there was an inter-trial interval of 0.9–1.4 s. The practice blocks used comparison durations that were 50%, 80%, 120%, and 150% to give participants exposure to some of the easier discriminations and allow them to learn the task, as well as some discriminations of medium difficulty as practice for the experimental block. There were 48 practice trials total where participants received feedback in the form of a tone if they selected the wrong stimulus. The main task comprised 540 trials for both the static and dynamic versions. For all blocks, the presentation order of the standard and comparison varied each trial.

### 2.4 Critical flicker frequency (CFF)

The CFF—defined as the frequency at which continuously flickering illumination is perceived as constant—was used as a marker of an individual’s temporal resolution in their vision (Cass et al., 2011). Temporal resolution is an aspect of visual processing that is often correlated with IAF in seemingly related tasks such as the two-flash fusion illusion (Cecere et al., 2015; Dou et al., 2022; Drewes et al., 2022; Keil & Senkowski, 2017; Migliorati et al., 2020; Samaha & Postle, 2015; Samaha & Romei, 2024; Venskus & Hughes, 2021). Individual differences in CFF are also related to IAF in clinical populations with hepatic encephalopathy (Baumgarten et al., 2018; Butz et al., 2013; May et al., 2014). We, thus, explored whether the CFF task might relate to IAF as well as the other duration perception measures in our non-clinical sample, and administered this task across both days of testing. The CFF was measured with the Flicker-Fusion system by Lafayette Instruments, which is designed to provide a quick (1–2 minutes) measure of an individual’s CFF. Participants viewed a flashing light through a viewing chamber and reported whether they perceived the light as flashing or continuous. The light was presented binocularly at the same rate to each eye, and the rate of presentation adaptively changed according to participant responses. The initial flicker frequency was 20 Hz, with initial changes of +/-5 Hz and final changes on the magnitude of +/- 0.1 Hz. Participants performed the task until the Flicker-Fusion system had identified the participant’s CFF.

### 2.5 Procedure

Each day, participants signed a written consent form and filled out some basic questions about their state that day (e.g., tiredness, hours since they last ate, etc.) before completing additional tasks. On day 1, participants answered demographic questions and completed the Comprehensive Autistic Trait Inventory (CATI) and Prodromal Questionnaire-Brief (PQ-B) questionnaires and the CFF task (see Supplemental Materials for more information). Participants then completed at least two practice blocks of each duration task (more if their performance was low or participants reported having difficulty with the task) followed by one experimental block of each task where there was no feedback. On day 2, participants quickly completed the CFF task before they were fitted with the EEG cap. They then completed one practice block and two experimental blocks of the duration estimation task, followed by one practice block and five experimental blocks of each duration discrimination task, with the static and dynamic task conditions counterbalanced. Finally, participants sat with their eyes closed for 2 minutes in order to collect resting-state data at the end of the session. Behavior and eyes-closed EEG data can be found at https://osf.io/5fc6q/overview. Participants were allowed to take breaks between blocks, as needed.

### 2.6 Behavioral data analysis

For the duration estimation task, we derived two standard measures of time estimation bias and precision by calculating each participant’s average estimates at each possible duration and their coefficient of variation (CV) in estimates at each possible duration, respectively. We calculated the CV for each participant and duration by dividing the standard deviation of participant estimates at each unique duration by the mean of their responses at each unique duration. The CV provided an unbiased measure of the variance in estimates, as it corrects for the larger variance inherent in estimates of longer durations. This measure can be interpreted as a precision measure (as variation in estimates decreases, precision increases). We also took the mean estimate and CV by collapsing across durations so that we had a single measure of estimation bias and precision for each participant.

Duration discrimination sensitivity was quantified from the discrimination tasks by fitting a logistic psychometric function (Palamedes toolbox version 1.10.4) to the proportion of times participants chose the comparison stimulus as “longer” for each of the possible comparison durations. This was done separately for each standard duration and each condition (static or dynamic). For all data fitting, the threshold parameter (reflecting the point of subjective equality, or PSE) was bounded by the range of comparison values, the slope parameter of interest (β) was set to -10 to 100 to capture the expected positive slope, and the “lapse rate” parameters (lambda and gamma) values were fixed at 0.05 to denote the lower and upper bounds. Slopes were not normally distributed, so we applied a log10 transformation to each slope parameter. Thus, we derived a slope parameter as a measure of sensitivity for each participant and condition (100 ms, 600 ms, and 1200 ms standards, static and dynamic conditions). Psychometric functions appeared to fit the duration discrimination data well for all participants and tasks except one participant who did not have a fit for the 1200 ms dynamic condition. To confirm this, we computed R-squared values for the fit for each participant, standard duration, and stimulus type. The average goodness of fit across all participants, durations, and stimulus types was 0.86 (SD = 0.16; range: 0.78–0.93). When averaging across tasks, only one participant—the same participant whose data we were unable to fit for one condition—had an average goodness-of-fit below 0.50. This participant was removed from subsequent analyses of discrimination task data.

### 2.7 EEG data collection

Continuous EEG was acquired from 63 active electrodes (BrainVision actiCHamp, iMotions A/S, Copenhagen, Denmark), with impedance kept below 20 kΩ. Recordings were digitized at 1000 Hz, and FCz was used as the online reference. EEG was processed offline using custom scripts in MATLAB (version R2019b) and using EEGLAB toolbox (Delorme & Makeig, 2004).

EEG signals were first high-pass filtered at 0.1 Hz, downsampled to 500 Hz, and re-referenced using a median reference (to avoid noisy channels contaminating the reference at this stage of pre-processing). Trials were epoched based on the onset time of the stimulus (or the first stimulus in the discrimination task) to include 2 s of prestimulus data and at least 800 ms of post-second-stimulus data based on the longest possible stimulus duration(s) for each task. We also epoched the eyes-closed resting data into 105 1 s-long epochs prior to manual inspection. All task data was then manually inspected to identify trials with muscular artifacts within a -500 prestimulus to +500 post-stimulus window, eye-blinks overlapping the stimulus presentations, and channels with excessive noise. After removing bad trials (estimation task: M = 14.62, SD = 19.83; discrimination task: M = 67.30, SD = 54.98) and interpolating noisy channels (estimation task: M = 2.71, SD = 1.76; discrimination task: M = 3.56, SD = 1.86) using spherical interpolation, an independent components analysis (ICA) was conducted, and ocular artifact components removed (M = 1.71, SD = 0.72). Eyes-closed data were manually inspected for noisy channels (M = 2.13, SD = 1.42) and epochs (M = 5.46, SD = 7.09) to be interpolated and rejected, respectively, but no ICA was run for this data. Finally, all data were average re-referenced and task data were baseline corrected using a 200 ms prestimulus baseline window.

### 2.8 Individual alpha frequency (IAF) analysis

**Fig. 2.**
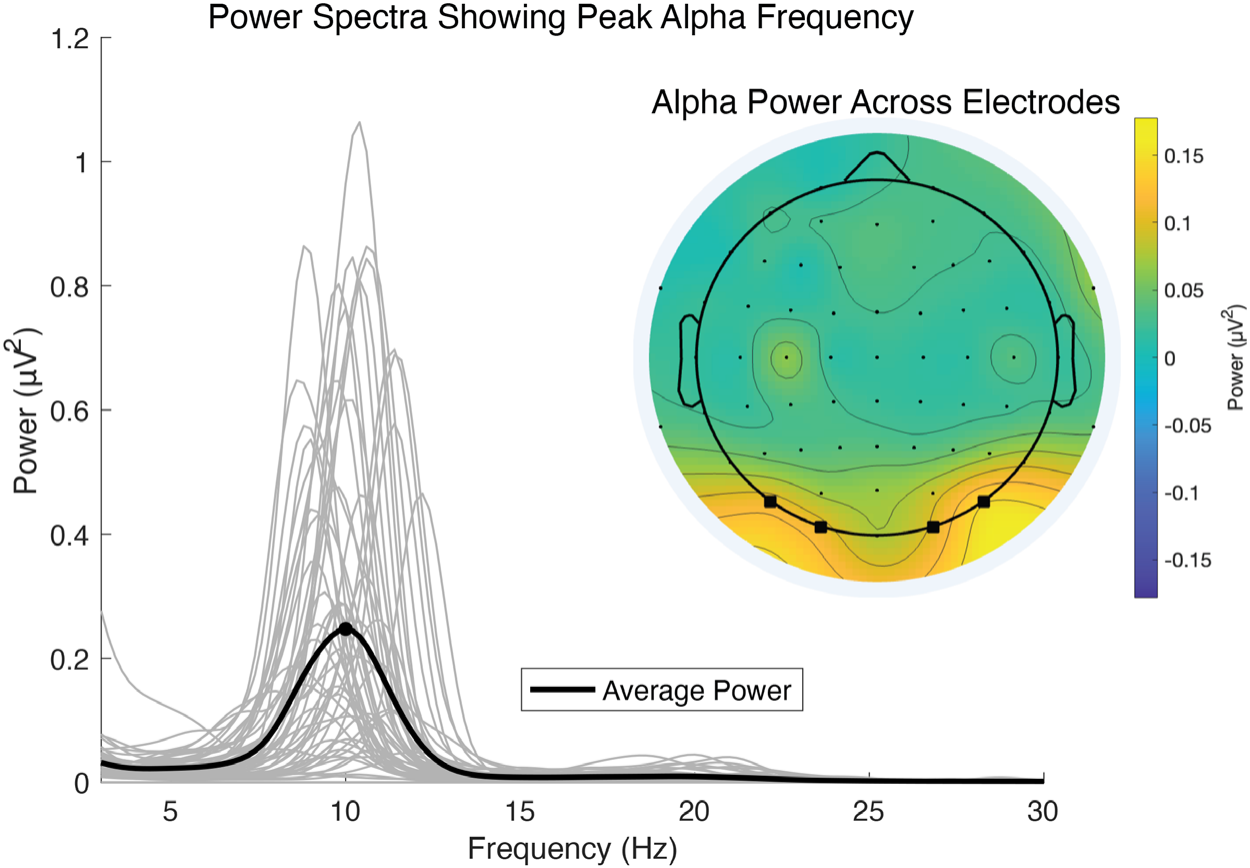
Individual and group power spectra from eyes-closed resting state data. The average power spectrum (black line) shows the average peak alpha frequency (black dot) is at 10 Hz. Gray lines are power spectra for individual subjects and emphasize the variability in peak frequencies. The topographical map shows the distribution of alpha power (7–14 Hz) across the scalp. Electrode locations (from left to right: PO7, O1, O2, PO8) with the highest group-level alpha power are highlighted with squares and were used in the FFT analysis to determine each individual’s peak alpha frequency.

A fast-Fourier transform (FFT) was used to identify peak alpha frequency for each individual from their eyes-closed resting data (Fig. 2). First, each 1 s epoch from the eyes-closed data was zero-padded, linearly detrended, and tapered using a Hamming window. After extracting power using the FFT, we averaged over an occipital electrode cluster (O1, O2, PO7, and PO8) with the highest group-level alpha power (see Fig. 2). We then used the MATLAB function *findpeaks.m* to identify the frequency within the alpha-band range (7–14 Hz) that had the highest power for each participant, resulting in their IAF. If participants did not have a clear alpha peak (n=1), they were assigned a peak of 10 Hz. Note that we did not apply a logarithmic transform to the power spectrum, which is sometimes done to emphasize the 1/f slope of the power spectrum or help normalize power values across trials (Gyurkovics et al., 2021). Since we were primarily interested in obtaining a peak frequency per participant, the lack of log-transformation of the spectra helps to emphasize the alpha peaks (see Fig. 2). However, the actual IAF values did not change as a result of this choice.

Our main goal was to evaluate the relationship between IAF and duration perception across individuals while controlling for participant age (which could correlate with IAF, performance, or both) and while examining the dependence of any effect on the actual stimulus duration. Thus, beginning with the duration estimation data, we ran two mixed-effects regression models using stimulus duration, IAF (mean-centered), age (mean-centered), and the interaction of stimulus duration and IAF to predict mean estimates and the CV of estimates. To examine specific relationships more directly, we also used Spearman correlations to assess the relationship between IAF and behavior in the duration estimation task (namely, the mean estimates and CV of estimates), after residualizing the behavioral measures for age. Finally, we verified the findings by performing a repeated-measures ANOVA (9 within-subjects durations, 2 between-subjects levels of IAF) to assess the effect of high versus low IAF, median split, on the estimation task measures.

For the duration discrimination task, we ran a mixed-effects regression model predicting duration discrimination slopes using the main effects of IAF (mean-centered), standard duration (100, 600, 1200), stimulus type (static or dynamic; dummy-coded), participant age (mean-centered), and the interactions between IAF and standard duration and IAF and stimulus type as predictors. To examine specific relationships from the model, we used Spearman correlations to assess the relationship between IAF and the slopes for each condition as well as the mean slopes across all conditions, after correcting for age in each behavioral measure of performance using a linear model. We also ran a repeated-measures ANOVA (2 within-subjects stimulus types, 2 between-subjects levels of IAF) on the duration discrimination data, median split by IAF.

### 2.9 Exploratory analysis

We were curious about the extent to which CFF related to IAF and duration perception sensitivity, as well as how the sensitivity measures across tasks related to one another. Given the non-normal distribution of CFF values (see Supplemental Figure 1), we computed separate Spearman correlations between IAF and the mean CFF and IAF and CFF from the second testing day, when IAF was measured. Additionally, we wanted to compare our main behavioral measures of interest across tasks to see how duration estimation related to duration discrimination sensitivity. We computed Spearman correlations between the mean slope of the duration discrimination task (averaged across conditions) and the CV of estimates (averaged across durations), as well as the mean slope of the duration discrimination task and the grand mean of estimates from the duration estimation task.

## 3 Results

### 3.1 Duration estimation

**Fig. 3.**
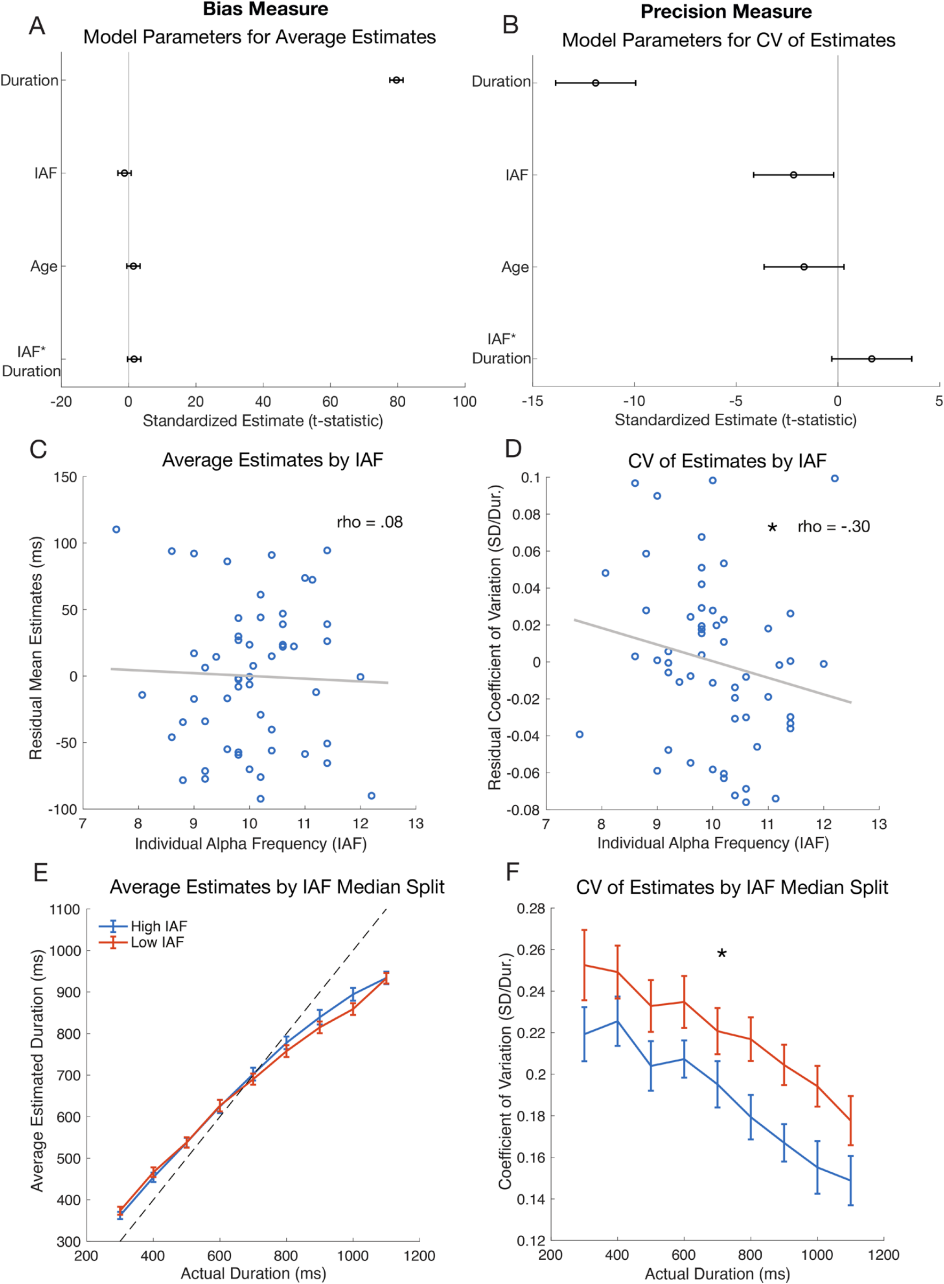
Duration estimation model output and measures, with average estimates (bias measures) plotted in the left column and the coefficient of variation (CV) of estimates (precision measures) plotted in the right column. (A-B) The effects (t-statistics and 95% CI) from linear mixed-effects models that were used to predict average estimates and CV of estimates using Stimulus Duration, IAF, Age, and the interaction term IAF * Stimulus Duration. (C-D) Average estimates and CVs of estimates were averaged across all durations and corrected for age. Plots show the correlation between IAF and the residuals for mean estimates and CV of estimates, where each blue dot indicates an individual participant. (E-F) Participants were split by the median IAF for visualization of the effect of IAF. Average estimates and CV of estimates are plotted for every actual duration that was presented in the duration estimation task. Participants with high IAF are shown in blue and participants with low IAF are shown in red (error bars are SEM). Asterisks indicate significance (*p* < .05).

Participants engaged in a duration estimation task where they observed a dot on each trial and input how long they estimated the dot to be on the screen for. We first examined participants’ average estimates at each duration (300–1100 ms, in 100 ms intervals) of the estimation task as a measure of bias (Fig. 3A, C, and E), and their coefficient variation around those estimates (CV) as a measure of precision (Fig. 3B, D, and F). Mean duration reports (Fig. 3E) were near veridical for medium durations (600–800 ms), were underestimated at longer durations (900–1100 ms), and overestimated at short durations (300–500 ms), replicating a well-documented pattern in time estimation and reproduction tasks thought to stem from a prior expectation over durations centered on the mean of the stimulus distribution (Jazayeri & Shadlen, 2010).

A mixed-effects model predicting mean estimates (Fig. 3A) demonstrated that stimulus duration was a significant predictor (β = 0.70, SE = 0.01, *t*(490) = 79.61, *p* < .001). As expected, as durations increased, so did the average duration estimates. No other predictors (age, IAF, or the interaction between IAF and duration) were significant in predicting mean estimation responses, suggesting IAF was not related to estimation bias. A mixed-effects model predicting the CV of estimates (Fig. 3B) found that CVs were significantly predicted by stimulus duration (β = -9.4 x 10^-5^, SE = 7.91 x 10^-6^, *t*(490) = -11.91, *p* = 6.75 x 10^-29^) and by IAF (β = -0.019, SE = 0.001, *t*(490) = -2.18, *p* = .03), but not by age or the interaction between IAF and duration. In other words, as durations increased, the CV of estimates decreased. Importantly, higher IAF was predictive of lower CV of estimates, indicating more precise time estimation responses. To help visualize this model, we also computed correlations between IAF and age-corrected behavioral measures. We found that age-corrected mean estimates (Fig. 3C) were not significantly related to IAF (*rho*(52) = .08, *p* = .57). However, the age-corrected CV of estimates (Fig. 3D) was significantly negatively correlated with IAF (*rho*(52) = -.30, *p* = .03), such that faster IAF was associated with a lower CV, or greater precision in one’s time estimation. Repeated-measures ANOVAs of estimation data split by IAF helped to confirm and visualize these results (Fig. 3E, F). We found a main effect of IAF split on the CV of estimates (*F*(1,53) = 6.92, *p* = .01). There was also a main effect of duration (*F*(1,53) = 1097, *p* = 4.29 x 10^-37^) and an interaction between duration and IAF split (*F*(1,53) = 7.42, *p* = .01), such that individuals with higher IAF had even greater precision at the long durations than individuals with lower IAF. We also found that IAF split interacted with duration to affect CV of estimates. For mean estimates, duration was a significant main effect (*F*(1,53) = 8502, *p* = 3.38 x 10^-60^), but IAF split and the interaction between IAF split and duration did not significantly affect mean estimates.

### 3.2 Duration discrimination

**Fig. 4.**
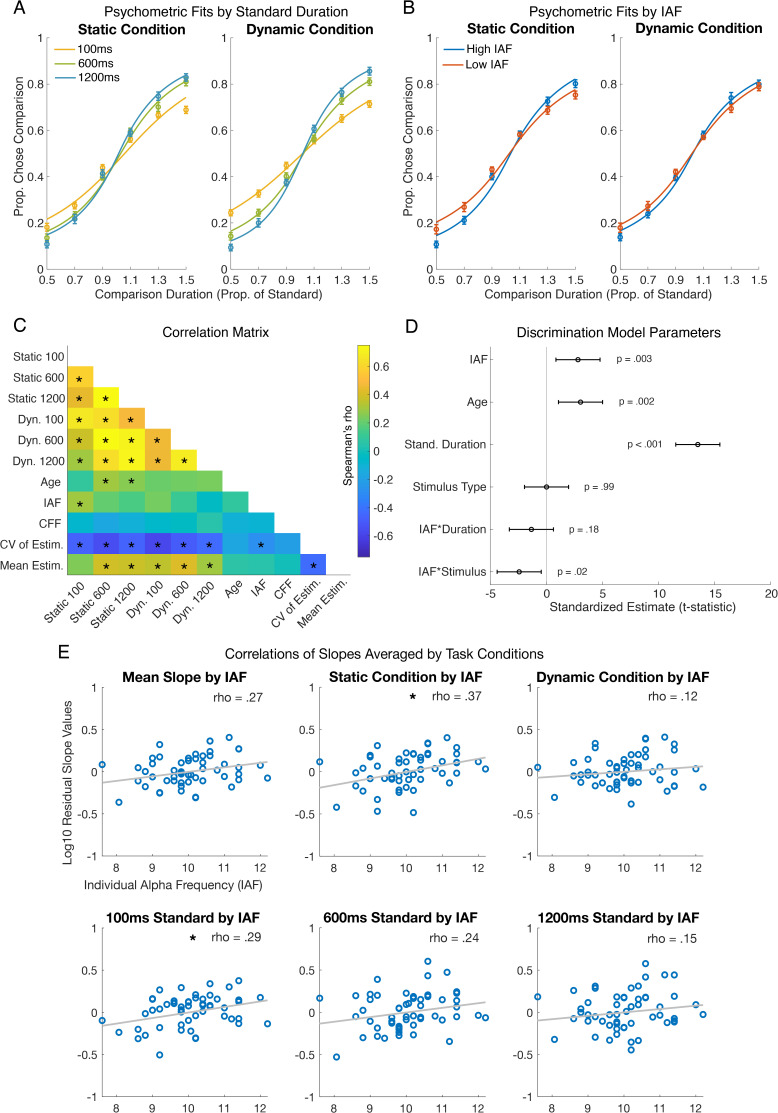
Duration discrimination results. (A) The proportion of times participants chose the comparison stimulus as being “longer” than the standard stimulus is plotted against the comparison duration (expressed in fractions of the standard). Psychometric function fits (lines) are shown alongside group mean data (circles with SEM error bars) for the 100 ms standards (yellow line), 600 ms standards (green line), and 1200 ms standards (blue line), separated by stimulus type. (B) Psychometric functions for participants with high IAF (blue line) and low IAF (red line) according to a median split shown separately for the static and dynamic tasks. (C) A correlation matrix showing the Spearman correlation between duration discrimination slopes from each condition (from top to bottom, or left to right: Static 100 ms, Static 600 ms, Static 1200 ms, Dynamic 100 ms, Dynamic 600 ms, Dynamic 1200 ms), Age, IAF, CFF, the CV of Estimates (the sensitivity measure in the estimation task), and the Mean Estimate (the bias measure in the estimation task). Note that the effects in the correlation matrix are not corrected for age. (D) The effects (t-statistics and 95% CI) from a linear mixed-effects model that was used to predict duration discrimination sensitivity (i.e., slopes) using IAF, Age, Standard Duration, Stimulus Type, and the interaction terms IAF * Standard Duration and IAF * Stimulus Type in the model. (E) Correlations between IAF and (age-corrected) slopes for different duration discrimination task conditions. The average discrimination slope across all conditions is plotted in the top left, with stimulus types plotted in the middle and top rightmost plots (static condition and dynamic condition, respectively). Slopes from each standard duration (averaged over static and dynamic conditions) are plotted in the bottom row (from left to right: 100 ms, 600 ms, 1200 ms). Each blue dot indicates an individual participant. Asterisks indicate significance (*p* < .05).

Participants also performed two duration discrimination task conditions (blocked and counterbalanced) where they discriminated the duration of either static or dynamic stimuli by reporting whether the first or second dot appeared longer in duration. Separate psychometric functions characterizing duration discrimination behavior were fit for each of the three standard durations (Fig. 4A) and for low and high IAF individuals based on median split (Fig. 4B). Performance in the discrimination task seemed to improve as durations became longer (Fig. 4A), despite the comparison durations being proportionally matched to satisfy Weber’s law (Haigh et al., 2021), implying a breakdown of Weber’s law in duration discrimination at the shortest (100 ms) duration. Still, we found that individual differences in slopes across all standard durations were positively correlated with one another, ranging from *rho*(53) of .26-.75; see Fig. 4C).

To estimate the joint effects of age, IAF, standard duration (100, 600, or 1200 ms), and stimulus type (static versus dynamic) on psychometric slopes using a single model that incorporates all the conditions of the discrimination task, we estimated a linear mixed-effects model (see Section 2 and Fig. 4D). We found that participant performance could be predicted using IAF, age, standard duration, and stimulus type (static or dynamic) as predictor variables (R^2^ = .65). Specifically, age (β = 0.01, SE = 0.005, *t*(317) = 3.14, *p* = .002), IAF (β = 0.09, SE = 0.03, *t*(317) = 3.02, *p* = .003), and standard duration (β = 2.83 x 10^-5^, SE = 2.12 x 10^-5^, *t*(317) = 13.44, *p* = 7.00 x 10^-5^) were significant predictors of slope as a measure of task performance. While stimulus type (static or dynamic) was not a significant predictor of performance, it significantly interacted with IAF to predict performance (β = -0.05, SE = 0.02, *t*(317) = -2.41, *p* = .02), indicating that the relation between the duration discrimination sensitivity and IAF was stronger for static compared to dynamic stimuli (as can be seen in Fig. 4B). There was no significant interaction between IAF and standard duration as a predictor variable, suggesting that the IAF relation to duration discrimination sensitivity was not strongly dependent on overall stimulus duration. Thus, when pooling together the discrimination sensitivity measures from all conditions into a single model, we found a clear positive association between IAF duration discrimination sensitivity that remained relatively constant across the three standard intervals tested (Fig. 4D). This effect indicates that individuals with higher IAF are more sensitive (i.e., have a steeper psychometric function slope) in discriminating between two different visual stimulus durations, and is confirmed by a significant main effect of IAF split (*F*(1,160) = 5.92, *p* = .02) in the repeated-measures ANOVA on overall duration discrimination performance (Fig. 4B). When not controlling for age or duration, there was a main effect of stimulus type (*F*(1,160) = 701.5, *p* = 2.25 x 10^-60^) and no effect of interaction between IAF split and stimulus type on performance.

To help unpack and visualize the model results, we next explored the correlations between IAF and age-corrected duration discrimination sensitivity for different groupings of the discrimination task conditions (Fig. 4E). First, we evaluated whether IAF was related to duration discrimination sensitivity when averaging over all durations and stimulus types. We found that condition-averaged sensitivity (slope) was moderately positively correlated with IAF (*rho*(51) = .27, *p* = .05), such that individuals with faster alpha frequency exhibited more sensitive time perception (steeper psychometric function slopes, after controlling for age). Breaking down this relationship further, we found a significant correlation between IAF and the average slope for the static condition (*rho*(51) = .37, *p* < .01), but not for the dynamic condition (*rho*(51) = .12, *p* = .41), consistent with the interaction effect observed in the mixed-effects model (Fig. 4D). Finally, there was a significant correlation between IAF and sensitivity discriminating 100 ms standards (*rho*(51) = .29, *p* = .03) and a marginal correlation between IAF and sensitivity discriminating 600 ms standards (*rho*(51) = .24, *p* = .08), but no significant relationship was found for the 1200 ms standards (*rho*(51) = .15, *p* = .29). Note, however, that these correlations are not statistically different from one another as reflected in the lack of a significant IAF-by-duration interaction in the mixed-model (Fig. 4D).

### 3.3 Exploratory results

We explored potential correlations between IAF with the mean CFF and day 2 CFF, and neither CFF score was significantly correlated with IAF (average CFF: *rho*(53) = -.09, *p* = .51; day 2 CFF: *rho*(53) = -.15, *p* = .26). Additionally, we examined whether performance on the different discrimination tasks (estimation versus discrimination) was related across individuals. Since the CV derived from duration estimates and the slope measure derived from 2IFC discrimination are both sensitivity measures, we would expect a relationship. Indeed, we found that the mean CV and mean slope of the psychometric fits were significantly negatively correlated across participants (*rho*(523) = -.546, *p* < .001; Fig. 4C), indicating that an individual with a steeper discrimination slope has lower estimation variability. Interestingly, there was also a relationship with the bias measure derived from the estimation task, such that participant’s mean overall estimates correlated significantly with the mean slopes of the psychometric fits (*rho*(523) = .461, *p* < .001).

## 4 Discussion

This study measured individual differences in sensitivity and bias in duration perception using both discrimination and estimation tasks across a range of peri-second durations spanning 100 ms to 1200 ms. Our key finding is that IAF shows a medium-sized correlation with time perception sensitivity as measured by both estimation and discrimination tasks, and was not found to predict measures of duration bias (i.e., under- or over-estimation). Importantly, these two sensitivity measures were significantly related: less variance in estimates was associated with a steeper psychometric function slope, highlighting a relationship between duration perception tasks that has not yet been explored. Moreover, our effects hold when controlling for participant age, a confound that may have influenced the conclusions in prior research. Our duration discrimination findings are consistent with, and expand upon, the vast literature supporting the role of IAF in sensitivity discriminating quick successive visual and multisensory stimuli (Cecere et al., 2015; Cooke et al., 2019; Di Gregorio et al., 2022; Migliorati et al., 2020; Noguchi, 2022; Samaha & Postle, 2015; Venskus & Hughes, 2021), and our duration estimation findings provide novel evidence that IAF also plays a role in the overall precision of estimating the duration of visual events.

By exploring the relation between IAF and a range of mostly sub-second durations, we show that IAF predicts time perception sensitivity approximately equally across the tested range, both in estimation and discrimination tasks. The shorter stimulus durations tested in our study were within what is traditionally considered closer to the “perceptual” side of duration judgment. The fact that our strongest correlation was found with 100 ms standard, where Weber’s law was observed to break down, reinforces the idea that the alpha frequency supports “short” duration discrimination which likely relies even more on sensory mechanisms such as those involved in temporal resolution and temporal binding of stimuli. That said, we still found effects of IAF on sensitivity at our longer durations, which is supported by prior findings (Mokhtarinejad et al., 2024).

The role of IAF in duration discrimination was, however, found to be specific to the static stimulus condition, as supported by a significant interaction between IAF and stimulus type in our mixed effects model. In our dynamic stimulus condition, we randomly modulated the luminance of the stimulus according to a white noise (equal energy at all frequencies) sequence. We anticipated this dynamic stimulus might enhance any link between IAF and duration discrimination; it may, instead, have entrained a broader range of frequencies and thus diluted the effect of alpha relative to the static condition. It’s also possible these stimuli were more cognitively demanding to observe, and duration perception in this task may have recruited more cognitive mechanisms. Alternatively, participants may have used different strategies for the dynamic task conditions, such as trying to count notable luminance changes. Due to the nature of our task conditions, the static and dynamic stimuli were not directly compared and thus we are unable to evaluate any potential biases towards the temporal perception of one stimulus type over another. This could be a compelling area of future research, particularly that of IAF in the context of the filled-duration effect, which has been seen across a number of modalities in behavioral duration perception studies (Buffardi, 1971; Hasuo et al., 2014; Wearden et al., 2007; Williams et al., 2019). Overall, it seems that the role of IAF in time perception may depend on the type of stimulus for which duration is being estimated, and additional stimulus properties should be explored.

The final analysis examining the association between IAF and CFF showed no significant relationship. Research on patients with hepatic encephalopathy shows that IAF correlates with CFF (Baumgarten et al., 2018; May et al., 2014). However, CFF and IAF may be uniquely correlated in these clinical populations, perhaps due to other underlying mechanisms that drive these patients to have lower IAF on average compared to healthy controls (Butz et al., 2013; Götz et al., 2013; May et al., 2014). The lack of correlation between IAF and CFF does fit with a recent paper that replicated the correlation between IAF and another measure of temporal resolution (the two-flash fusion paradigm) but not with CFF, suggesting these two measures tap different aspects of temporal resolution (Haarlem et al., 2024).

Our main results contradict some recent results whereby IAF and alpha phase did not relate to sensitivity (Milton & Pleydell-Pearce, 2016; Mioni et al., 2020). However, both tasks used a similar standard duration (400 ms or 600 ms) which was either learned before viewing comparisons, or always presented as the first stimulus. Perceptual training research (Navarra et al., 2005; Powers et al., 2009; Stevenson et al., 2013) and research on the temporal order effect (Grondin, 2010) suggest that these design considerations have important implications for how participants may have performed on those duration tasks. Additionally, the lack of sensitivity effect when altering IAF with tACS supports the idea that we may be calibrated to the sensitivity supported at our IAF, and not other alpha frequencies or sampling rates. Importantly, research using similar discrimination designs does indeed support the role of alpha frequency in sensitivity (Frisoni et al., 2025), even showing a role of alpha power in accuracy (Mokhtarinejad et al., 2024). While the prior research is conflicting, we note that our sample size was considerably larger than those prior papers and we found consistent results across two distinct time perception tasks and across different stimulus durations. However, it could still be valuable to evaluate performance on these temporal perception tasks in relation to other alpha characteristics which might be underlying perceptual biases, such as phase, alpha bursts, or changes in alpha power, as seen in the prior literature (Azizi et al., 2023; Cahoon, 1969; Legg, 1968; Milton & Pleydell-Pearce, 2016; Mokhtarinejad et al., 2024; Werboff, 1962).

Our findings can be interpreted in the theoretical context of both intrinsic and dedicated timing mechanisms. An association between IAF and bias in duration perception would have more clearly supported the role of the dedicated pacemaker-accumulator model (Treisman et al., 1990), given that this model assumes that a specific chronotopic mechanism is consistently governing timing, whereas the intrinsic model suggests timing is governed by the more variable interactions between ongoing neural dynamics and sensory inputs (Ivry & Schlerf, 2008). The finding of a correlation with sensitivity, on the other hand, less clearly distinguishes between theories. The association we found between IAF and sensitivity indicates that alpha cycles moderate the strength or fidelity of perceptual moments, which could support intrinsic timing mechanisms that rely on the dynamics of sensory processing. However, sensitivity could also be attributed to dedicated mechanisms, if the accumulation of perceptual moments, per the pacemaker-accumulator model, results in higher overall fidelity. Future research is needed to distinguish these mechanisms, particularly as more time perception research seems to indicate support for the link between alpha and sensitivity (Frisoni et al., 2025; Mokhtarinejad et al., 2024). Aligned with these more recent findings, much of the literature on temporal binding windows of visual perception suggests IAF relates to sensitivity on perceptual tasks (for a meta-analysis, see Samaha & Romei, 2024), although other research has challenged these findings (Buergers & Noppeney, 2022), and still others have theorized that alpha moderates perception discretely and introduces perceptual bias instead (Schneider, 2018; VanRullen, 2016).

We note that many important future directions stem from this work, and could help to further our understanding of how alpha governs time perception. The dedicated model suggests a pacemaker may operate locally or centrally, while the intrinsic model seems to suggest that the unique neural dynamics govern timing in the different sensory cortices (Ivry & Schlerf, 2008; Mauk & Buonomano, 2004). Thus, additional oscillatory features and frequencies, and other tasks and modalities, should be evaluated in the context of duration perception. Examining whether IAF plays a role in perceiving durations for non-visual stimuli is crucial to establish. Research suggests that frequencies outside of the alpha-band range are associated with duration perception in motor and auditory processing (Botcharova et al., 2015; Luo & Poeppel, 2012; Uehara et al., 2023), and that these processes differ at the behavioral level (Ortega et al., 2014; Tomassini et al., 2011; Wearden et al., 2007). These findings imply a likelihood that separate neural dynamics govern timing in each modality, but should be tested more specifically. Additionally, given the strong role of IAF in cross-modal binding (Cecere et al., 2015; Keil & Senkowski, 2017; Migliorati et al., 2020; Venskus et al., 2021), and some evidence that alpha phase modulates auditory and tactile detection (Ai & Ro, 2014; Rice & Hagstrom, 1989; Thorne et al., 2011), more research is needed to rule out alpha’s role as a central pacemaker. Time perception research in other modalities would further our understanding of how the different frequency bands contribute to timing across the senses.

Overall, our results support the idea that alpha rhythmically drives sensitivity in time perception, as the theory would suggest that individuals with faster IAF have more frequent excitatory perceptual windows and therefore more frequent updating of visual information to be used for estimating stimulus duration. Further bolstering this interpretation of our findings is the fact that the sensitivity measures (CV of estimates and slope in discriminations) were significantly negatively correlated across tasks: as discrimination sensitivity increased, the amount of variance in estimates decreased. While this correlation is not sufficient to conclude that these processes are supported by the same underlying mechanism, it is a necessary relationship to observe if IAF is, indeed, the mechanism driving the magnitude of sensitivity across participants in both visual duration perception tasks. Critically, though, causal methods are needed to establish the mechanistic role of alpha oscillations in time perception.

## Supplementary Material

Supplementary Material

## Data Availability

The behavioral data and cleaned resting-state EEG data, as well as scripts for analyzing task data and extracting IAF, can be found at https://osf.io/5fc6q/overview.
