# Supplementary material for: Individual alpha frequency predicts the sensitivity of time perception
Source: Imaging Neurosci (Camb). 2026 Jun 4;4:IMAG.a.1263. doi: 10.1162/IMAG.a.1263 (PMC13237996; doi:10.1162/IMAG.a.1263)
Supplement: Supplementary Material [file IMAG.a.1263_supp.pdf]

Supplemental Materials

A meta-analysis suggests that individuals with schizophrenia are less accurate at discriminating durations and have the tendency to overestimate durations (Thoenes & Oberfeld, 2017). However, despite evidence that some individuals with ASD have atypical sensory processing, characterized by improved perception of local features (detail), and impaired perception of global structure (contextual information) (Chung & Son, 2020; Dakin & Frith, 2005), there is conflicting evidence around whether individuals with ASD have typical or enhanced processing of time intervals (Poole et al., 2022; Wallace & Happé, 2008). Thus, we conducted exploratory analyses to assess whether IAF or duration perception performance related to questionnaire scores measuring Autistic-like traits and prodromal (schizotypal) experiences in each participant.

To measure autistic traits in our college sample, we used the CATI (English et al., 2021), a 42-item inventory that asks participants to rate how much they agree with statements associated with traits typically seen in the ASD population. Responses are given via a 5-point scale ranging from “Definitely Disagree” to “Definitely Agree”. The items come from one of six main categories of traits (Social Interactions, Communication, Social Camouflage, Cognitive Rigidity, Repetitive Behavior, and Sensory Sensitivity) and include statements like, “Metaphors or ‘figures of speech’ often confuse me,” and “I feel discomfort when prevented from completing a particular routine.” Answers for each of the items were totaled for each participant, providing a score between 42-210.

The PQ-B (Loewy et al., 2011) was used to measure schizotypal or prodromal traits in the neurotypical population, an inventory consisting of 21 items asking about thoughts, feelings, and experiences within the past month. Items were responded to with a “yes” or “no” and included statements such as, “Do familiar surroundings sometimes seem strange, confusing, threatening or unreal to you?” and “Have you felt that you are not in control of your own ideas or thoughts?”. If the participant responded “yes”, a follow-up distress scale item asked whether the experience caused the participant to feel “frightened, concerned, or it causes problems” on a 5-point scale ranging from “Strongly Disagree” to “Strongly Agree”. Participant scores were computed in two ways: 1) by totaling the number of “yes” responses to get a score between 0-21, and 2) by totaling the values of the distress scale responses to get a score between 21-105. We were primarily interested in the first score which provided a numerical range of the prodromal experiences across individuals.

Given our non-clinical sample, we found generally low CATI and PB-Q scores (Supplemental Figure 1A). Critical flicker frequency (CFF) scores, which research suggests is related to IAF in clinical populations (Baumgarten et al., 2018; Butz et al., 2013; May et al., 2014), was also somewhat skewed in our sample (Supplemental Figure 1A). The range of scores for the Comprehensive Autistic Trait

Inventory (CATI) was 73-187 (M = 116.16, SD = 26.50), and the range for the Prodromal Questionnaire-Brief (PQ-B) was 0-18 (M = 4.89, SD = 4.45).

No significant correlation was found between IAF and individual's CATI scores or PQ-B scores. The CATI and PQ-B scores also did not significantly relate to any of the main performance measures of interest (mean estimates, CV of estimates, and duration discrimination slopes). We also evaluated whether the CFF from the first day, the testing session where participants completed the trait questionnaires, was correlated with either questionnaire score (Supplemental Figure 1B). We found a moderately weak, non-significant, correlation between CFF and CATI scores ($rho(53) = .24$, $p = .08$) and PQ-B scores ($rho(53) = .23$, $p = .09$). Overall, there were no significant correlations between IAF and the magnitude of ASD traits (CATI) or prodromal traits (PB-Q) in our population, perhaps due to the non-normal distribution of trait scores, whereby individuals tended to score low in both of these traits (Supplemental Figure 1B). While we found lower IAF to be associated with reduced duration perception sensitivity, it does not seem to be directly related to ASD or prodromal traits in normal healthy adults.

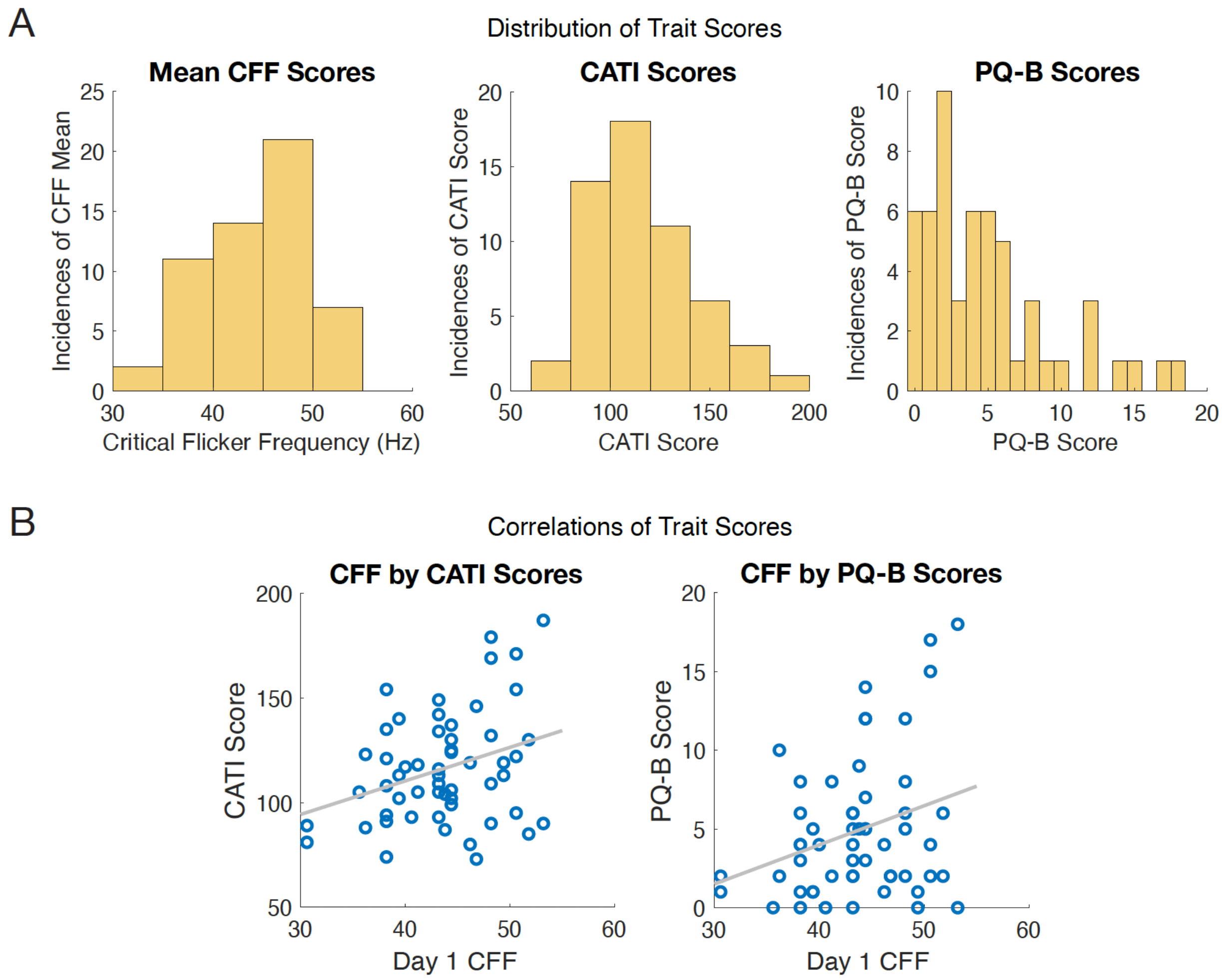

Supplemental Figure 1. Participants were asked to complete two questionnaires, the Comprehensive Autistic Trait Inventory (CATI) (English et al., 2021) and the Prodromal Questionnaire-Brief (PQ-B) (Loewy et al., 2011) to assess the extent to which autistic and prodromal schizotypy traits in a typical college sample related to variations in IAF and duration perception, as well as the critical flicker frequency (CFF) task, across both days of the study. (A) Histograms demonstrate the number of participants with each mean CFF (left) and total CATI (middle) and PQ-B (right) scores across the sample. (B) Results from Spearman correlations that were computed between the Day 1 CFF scores and CATI scores (left) and PQ-B scores (right) as an exploratory measure of the relationship between traits that were expected to, but did not relate to, IAF. Neither correlation reached statistical significance.

References

Baumgarten, T. J., Neugebauer, J., Oeltzschner, G., Füllenbach, N.-D., Kircheis, G., Häussinger, D., Lange, J., Wittsack, H.-J., Butz, M., & Schnitzler, A. (2018). Connecting occipital alpha band peak frequency, visual temporal resolution, and occipital GABA levels in healthy participants and hepatic encephalopathy patients. *NeuroImage: Clinical*, *20*, 347–356. https://doi.org/10.1016/j.nicl.2018.08.013

Butz, M., May, E. S., Häussinger, D., & Schnitzler, A. (2013). The slowed brain: Cortical oscillatory activity in hepatic encephalopathy. *Archives of Biochemistry and Biophysics*, *536*(2), 197–203. https://doi.org/10.1016/j.abb.2013.04.004

Chung, S., & Son, J.-W. (2020). Visual Perception in Autism Spectrum Disorder: A Review of Neuroimaging Studies. *Journal of the Korean Academy of Child and Adolescent Psychiatry*, *31*(3), 105–120. https://doi.org/10.5765/jkacap.200018

Dakin, S., & Frith, U. (2005). Vagaries of Visual Perception in Autism. *Neuron*, *48*(3), 497–507. https://doi.org/10.1016/j.neuron.2005.10.018

English, M. C. W., Gignac, G. E., Visser, T. A. W., Whitehouse, A. J. O., Enns, J. T., & Maybery, M. T. (2021). The Comprehensive Autistic Trait Inventory (CATI): Development and validation of a new measure of autistic traits in the general population. *Molecular Autism*, *12*(1), 37. https://doi.org/10.1186/s13229-021-00445-7

Loewy, R. L., Pearson, R., Vinogradov, S., Bearden, C. E., & Cannon, T. D. (2011). Psychosis risk screening with the Prodromal Questionnaire—Brief Version (PQ-B). *Schizophrenia Research*, *129*(1), 42–46. https://doi.org/10.1016/j.schres.2011.03.029

May, E. S., Butz, M., Kahlbrock, N., Brenner, M., Hoogenboom, N., Kircheis, G., Häussinger, D., & Schnitzler, A. (2014). Hepatic encephalopathy is associated with slowed and delayed stimulus-associated somatosensory alpha activity. *Clinical Neurophysiology*, *125*(12), 2427–2435. https://doi.org/10.1016/j.clinph.2014.03.018

Poole, D., Casassus, M., Gowen, E., Poliakoff, E., & Jones, L. A. (2022). Time perception in autistic adults: Interval and event timing judgments do not differ from nonautistics. *Journal of Experimental Psychology: General*, *151*(11), 2666–2682. https://doi.org/10.1037/xge0001203

Thoenes, S., & Oberfeld, D. (2017). Meta-analysis of time perception and temporal processing in schizophrenia: Differential effects on precision and accuracy. *Clinical Psychology Review*, *54*, 44–64. https://doi.org/10.1016/j.cpr.2017.03.007

Wallace, G. L., & Happé, F. (2008). Time perception in autism spectrum disorders. *Research in Autism Spectrum Disorders*, *2*(3), 447–455. https://doi.org/10.1016/j.rasd.2007.09.005
